# Facteurs de risque et pronostic materno-fœtal de la macrosomie fœtale: étude comparative a propos de 820 cas Risk factors and materno-fetal prognosis of foetal macrosomia: comparative study of 820 cases

**DOI:** 10.11604/pamj.2017.28.126.8508

**Published:** 2017-10-10

**Authors:** Fatnassi Ridha, Ragmoun Houssem, Marzougui Latifa, Mkhinini Ines, Hammami Sabra

**Affiliations:** 1 Département de Gynécologie Obstétrique, CHU Ibn El Jazzar, Kairouan, Tunisie; 2 Département d’Epidémiologie Chu Ibn El Jazzar, Kairouan, Tunisie

**Keywords:** Macrosomie, échographie, dystocie des épaules, déchirure périnéale, Macrosomia, ultrasound, shoulder dystocia, perineal tear

## Abstract

L’accouchement d’un fœtus macrosome est une situation relativement fréquente. Il s’agit d’un accouchement à haut risque aussi bien maternel que fœtal. Les principales complications maternelles étaient représentées par l’augmentation du taux de césarienne, d’hémorragie de la délivrance et des déchirures traumatiques cervico-vaginales. La principale complication fœtale était la dystocie des épaules exposant aux élongations du plexus brachial. L’objectif était d’identifier les facteurs de risque et les complications materno-fœtales liées à la macrosomie fœtale. Une étude rétrospective comparative effectuée au service universitaire de maternité de Kairouan durant l’année 2010. Au cours de cette étude, nous comparons un groupe de 820 cas de macrosomes à un groupe de 800 cas de témoins nés au cours de la même période. Au cours de la période d’étude, nous avons colligé 820 cas de macrosomies sur un total de 7495 naissances soit une incidence de 10.94%. Plusieurs facteurs favorisants la macrosomie fœtale étaient mis en évidence: un âge maternel >35 ans était présent dans 28.5% des cas; L’obésité maternelle était trouvée dans 45% des cas; Les antécédents de macrosomie étaient notés dans 28,8% des cas; Le terme prolongé > 41 semaines d’aménorrhée était noté dans 35.6% des cas; La multiparité était trouvée dans 47% des cas. Les complications maternelles étaient essentiellement l’hémorragie de la délivrance: 71 cas et les traumatismes génitaux: 24 cas. Les complications périnatales étaient dominées par la dystocie des épaules, 27 cas soit 3.3%. Les complications postnatales traumatiques étaient retrouvées dans 11.6%.

## Abstract (en)

The delivery of a macrosomic infant is a relatively common situation. It can put mother and fetus at high risk. The main maternal complications are the increase in cesarean rates, postpartum hemorrhage and cervicovaginal traumatic lacerations. The main fetal complication is shoulder dystocia increasing the risk of brachial plexus. The objective was to identify risk factors and maternal-fetal complications associated with fetal macrosomia. Comparative retrospective study conducted at Kairouan University Hospital maternity unit in 2010. We compared a group of 820 cases of macrosomic infants to a control group of 800 cases of infants born in the same period of time. During the study period we collected clinical data of 820 macrosomic cases on a total of 7.495 deliveries, corresponding to a total incidence of 10.94%. Several factors predisposing to fetal macrosomia were highlighted: Maternal age> 35 years was present in 28.5% of cases; Maternal obesity was found in 45% of cases; A personal history of macrosomia was noted in 28,8% of cases; Prolonged pregnancies > 41 weeks of amenorrhoea was noted in 35.6% of cases; Multiparity was found in 47% of cases. Maternal complications were essentially postpartum hemorrhage: 71 cases and genital traumas: 24 cases. Perinatal complications were dominated by shoulder dystocia: 27 cases (3.3%). Traumatic postpartum complications were found in 11.6%.

## Introduction

L’accouchement d’un fœtus macrosome est à haut risque materno-fœtal. En effet les fœtus peuvent être exposés à de nombreuses complications dont la plus fréquente est la dystocie des épaules [1, 2]. Ils peuvent garder des séquelles irréversibles secondaires à l’asphyxie fœtale et même décéder [3]. Les complications maternelles sont également graves. Elles peuvent survenir dans le per partum tels sont les cas des ruptures utérines et des déchirures périnéales, ou plus tardivement et sont à type de prolapsus uro-génital et d’incontinence urinaire ou anale. Si les études précédentes se sont intéressées aux taux de dystocie des épaules et des lésions traumatiques prénatales chez les macrosomes sans comparaison avec ceux observés chez les nouveau-nés eutrophiques, notre étude trouve son intérêt, en effet c’est une étude comparative ayant pour objectif de définir les facteurs de risque de la macrosomie, préciser les modes d’accouchement ainsi que les complications maternelles, fœtales et périnatales inhérentes à la macrosomie.

## Méthodes

Il s’agit d’une étude rétrospective comparative effectuée à la maternité Universitaire de Kairouan durant une période d’un an, allant du 01 janvier au 31 décembre 2010. Pendant cette période 7495 accouchements ont été effectués. La définition de la macrosomie que nous avons retenu pour ce travail est un fœtus de poids de naissance supérieur ou égal à 4000 g à terme. Alors que, Le groupe contrôle était composé de nouveaux nés vivants de poids de naissance compris entre 2500g et 3999g. Le groupe des macrosomes était composé de 820 nouveaux nés, le groupe contrôle comptait 800 nouveau nés. Nous avons collecté les renseignements concernant la grossesse actuelle et les grossesses précédentes ainsi que le devenir néonatal. Nous avons relevé également les complications du per partum et du post partum de 1620 cas, qui ont été extraites des registres du suivi prénatal et des dossiers d’accouchement.

***Les critères d’inclusion:*** Sont retenues dans cette étude les grossesses uniques et à terme lors de l’accouchement (âge gestationnel > à 37 SA).

***Les critères de non inclusion:*** Ont été exclus de ce travail les fœtus présentant une anomalie chromosomique ou somatique ainsi que les accouchements à domicile, les grossesses multiples et les MFIU quelque soit le poids de naissance.

Les études statistiques ont été accomplies à l’aide des tests de X2 et t de Student, en utilisant le logiciel SPSS (version 13). Une valeur de p < 0,05 était considérée comme significative.

## Résultats

**Figure 1:**
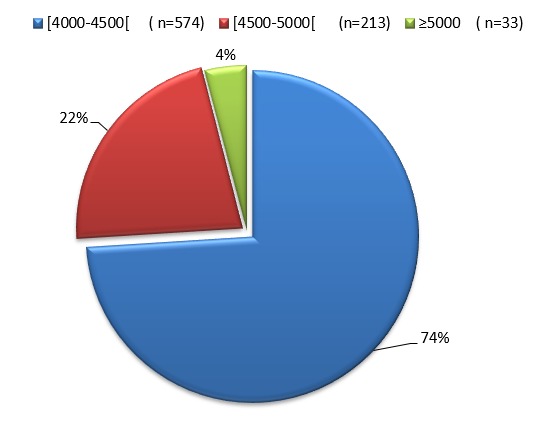
La répartition du poids de naissance des macrosomes

Sur un total de 7495 accouchements effectués durant l’année 2010, nous avons relevé 820 naissances macrosomes. Soit une fréquence de macrosomie fœtale de 10,94%. La répartition du poids de naissance dans notre population de macrosomes avait montré que les ¾ du groupe étudié avaient un poids entre 4000 et 4500 g (Figure 1): le sexe ratio (M/F) chez les macrosomes était de 1,7 Par contre dans le groupe contrôle il était de 1,29 (Cette différence est significative avec X2 = 9,3 et P < 0,01).

### Caracteristiques maternelles

**Figure 2:**
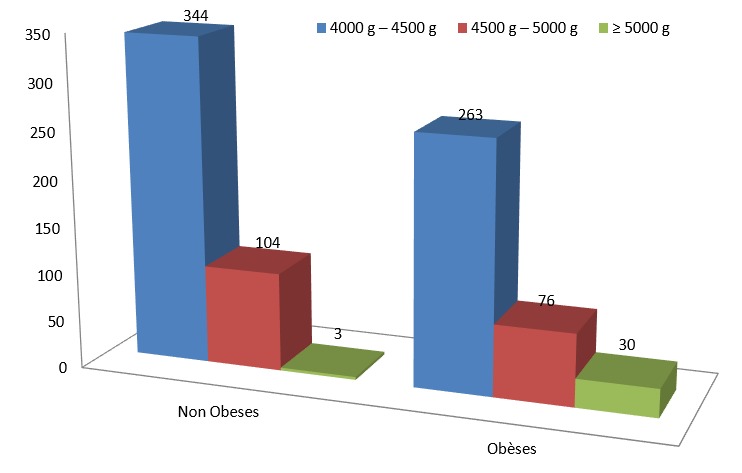
Répartition du poids de naissance des macrosomes en fonction de L’IMC maternel

Les caractéristiques maternelles et les antécédents obstétricaux sont représentés dans le Tableau 1. Une obésité était constatée chez 45% des patientes du groupe d’étude contre 18% dans le groupe témoin. La différence était significative (P<0,001) (Figure 2), par ailleurs, la prise de poids maternelle n’a pas pu être évaluée dans notre étude car on ne disposait pas du poids pré gravidique de nos patientes.

**Tableau 1:** Caractéristiques maternelles et pathologies gravidiques

		Groupe Macrosome	Groupe Control	p
Caractéristiques de la mère	L’âge moyen (année)	31,51	30,32	0.1
	La gestité moyenne	2,8	2,2	-
	La parité moyenne	2,7	2,1	-
	Multiparité	385 (46,9%)	268 (33,5%)	<0,001
	Taille > 1,6 m	455 (55,5%)	362(45,25%)	0,02
	Le poids moyen	86,4	74,5 kg	<0,001
	l’obésité	369 (45%)	144 (18%)	<0,001
Antécédents obstétricaux	Antécédents de macrosomie	236(28,8%)	142(17,75%)	<0,001
	Antécédent de MFIU	18(2,2%)	6(0,75%)	<0,02
	Antécédent de mort néonatale	42 (5,1%)	19(2,4%)	<0,01
Pathologies gravidiques	Diabète gestationnel	61 (7,4%)	34 (4,2%)	0,01
	Pré éclampsie	51 (6,2%)	55 (6,9%)	0,9
	MAP	19 (2,3%)	21 (2,6%)	0,9
	Hydramnios	11 (1,3%)	2 (0,2%)	0,02
	Pyélonéphrite aiguë	8 (0,9%)	6 (0,7%)	0,9
	Autres	18 (2,2%)	15 (1,9%)	0,8

Dans notre étude, les pathologies gravidiques étaient représentées essentiellement par le diabète gestationnel, la pré-éclampsie, la menace d’accouchement prématuré, l’hydramnios et la pyélonéphrite aiguë gravidique. 168 patientes du groupe étudié soit 20,5% avaient présenté une pathologie gravidique contre 133 patientes dans le groupe témoin soit 16,6%.

### Caracteristiques de la grossesse et l’accouchement (Tableau 2)

Le terme moyen d’accouchement dans le groupe des macrosomes était de 40,2 semaines tandis qu’il était de 39,6 semaines dans le groupe contrôle. Les principales indications du déclenchement artificiel du travail étaient la pré éclampsie (46 cas chez les macrsomes, 31 contrôle), le diabète (41 cas chez les macrsomes, 25 contrôle) et le dépassement de terme (37 cas chez les macrsomes, 23 contrôle). La Stagnation de la dilatation (p=0,01) et l’échec du déclenchement (p=0,02) étaient les deux indications de césarienne qui avaient une différence statistiquement significative entre les deux groupes.

**Tableau 2:** Caractéristiques de la grossesse et modes d’accouchement

		Macrosomes N (%)	Contrôle N(%)	p
Le terme	prolongé	292 (35,6)	215 (26,9)	< 0,001
	Dépassement	83(10,1)	46(5,7)	< 0,01
L’hydramnios		11(1,3)	2(0,2)	0,02
Mode d’entrée en travail	Travail spontané	536 (65,4)	612 (76,5)	< 0,01…
	Déclenchement artificiel	162 (19,7)	104 (13)	0,02…
Présentations Fœtales	Sommet	780 (95,1%)	771 (96,5%)	NS
	Siège	32 (3,9%)	24 (3%)	NS
	Autres	8(0,1)	5(0,5)	NS
Travail	Phase de latence > 4h	541(79)	414(59,3)	<0,001
	Phase active > 3h	522(76,3)	374(374)	< 0,001
	Césarienne au cours du travail	66(8)	36(4,5)	NS
	Durée expulsion > 30 mn	476(5,8)	144(1,8)	< 0,001
Mode d’accouchement	voie basse (VB)	572(69,7)	634(79,2)	0,001
	(VB)+aide instrumentale	46(5,6)	28(3,5)	0,02
	césarienne	202(24,6)	138(17,25)	< 0,001

Quant aux indications d’extraction instrumentale, elles se répartissaient comme suit: 1) 36 cas pour défaut d’expulsion soit 78,2%; 2) Un épuisement maternel dans 8 cas, soit 17,4%; 3) Une souffrance fœtale aigue dans 2 cas, soit 4,3%; 4) Seule l’indication d’une césarienne élective pour un PFE supérieur ou égal à 4500 g était significativement plus fréquente dans le groupe des macrosomes que dans le groupe témoin (Tableau 3).

**Tableau 3:** Indications des césariennes prophylactiques dans notre série

Groupes Indications de la C/S élective	Macrosomes n(%)	Contrôle n(%)	P
PFE ≥ 4500 g	46 (33,8)	4 (3,9)	< 0,001
Utérus uni cicatriciel	30 (22)	28 (27,4)	0,4
Présentation de siège	22 (16,8)	17 (16,6)	0,9
Bassin limite	12 (8,8)	18 (17,6)	0,04
Utérus bi cicatriciel	9 (6,6)	13 (12,7)	0,1
Pré éclampsie sévère	8 (5,8)	8 (7,8)	0,6
Diabète déséquilibré	6 (4,4)	8 (7,8)	0,3
Autres	3 (2,2)	6 (5,9)	0,2
Total	136	102	

### Morbidité et mortalité maternelle

Dans notre étude, aucun décès maternel n’était déploré. Toutefois, nous avons relevé 140 cas de complications maternelles suite à des accouchements de nouveau-nés macrosomes soit un taux de 17% contre 113 cas de complications maternelles dans le groupe témoin soit un taux de 14,1%. La morbidité maternelle dans le groupe des macrosomes était nettement supérieure à celle dans le groupe témoin. Elle était dominée par la pathologie traumatique, les hémorragies de la délivrance et les complications infectieuses, mais aucune rupture utérine n’était enregistrée (Tableau 4).

**Tableau 4:** Morbidité maternelle et fœtale

	Macrosomes		Témoins	P
Morbidité maternelle (césarienne)	Atonie utérine	9 (1,1%)	5 (0,6%)	0,9
	Lésion du segment inférieur	8 (0,97%)	4 (0,5%)	0,9
	Endométrite post opératoire	6 (0,7%)	4 (0,5%)	0,9
	Abcès de la paroi	6 (0,7%)	4 (0,5%)	0,9
	Hématome de la paroi	3 (0,4%)	2 (0,25%)	0,9
	Infection urinaire	6 (0,7%)	5 (0,6%)	0,9
	Total	38 (4,6%)	24 (3%)	0,9
Morbidité maternelle (voie basse)	Hémorragie de la délivrance	71 (8,6%)	63 (7,9%)	0,3
	Déchirure périnéale	17 (2,1%)	14 (1,7%)	0,5
	Déchirure cervicale	7 (0,8%)	4 (0,5%)	0,5
	Infection de l’épisiotomie	7 (0,8%)	8 (1%)	0,9
	Total	102 (12,4%)	89 (11,1%)	0,2
Morbidité fœtale Périnatale	Traumatisme obstétrical	95 (11,6%)	62 (7,7%)	0,01
	Souffrance fœtale aiguë	37 (4,5%)	29 (3,6%)	0,5
	Détresse respiratoire néonatale	26 (3,7%)	16 (2%)	0,2
	Hypoglycémie	22 (2,7%)	10 (1,2%)	0,05
	Total	180 (21,9%)	117 (14,5%)	<0,001
Traumatismes Néonataux	Bosse séro-sanguine	65 (66,3%)	58 (77,3%)	0,7
	Ecchymose de la face	7 (7,1%)	5 (6,7%)	0,7
	Hématome sous cutané	5 (5,1%)	4 (5,3%)	0,9
	Paralysie du plexus brachial	13 (13,3%)	6 (8%)	0,2
	Paralysie faciale	3 (3%)	2 (2,7%)	0,8
	Fracture de la clavicule	5 (5,1%)	0 (0%)	0,04
	Total	98 (100%)	75 (100%)	

### Morbidité périnatale

Dans notre étude nous avons recensé 180 cas de complication périnatale à la suite d’accouchement de nouveau-nés macrosomes soit un taux de 21,9% contre 117 cas de complications périnatales dans le groupe témoin soit un taux de 14,5%. La différence était significative (P < 0,001). Les principales complications néonatales enregistrées étaient: 1) 95 cas de traumatisme obstétrical soit une fréquence de 11,8%; 2) 37 cas de souffrance fœtale aiguë soit 4,5% des cas; 3) 26 cas de détresse respiratoire néonatale soit une fréquence de 3,2%; 4) 22 cas d’hypoglycémie soit une fréquence de 2,7%. Les étiologies des détresses respiratoires néonatales dans le groupe des macrosomes étaient de type transitoire dans 10 cas, par inhalation méconiale dans 9 cas et suite à une infection materno-fœtale dans 7 cas.

### Dystocie des épaules

Dans notre étude, la dystocie des épaules avait compliqué 27 accouchements dans le groupe des macrosomes soit 3,3% de tous les accouchements et 4,4% des accouchements par voie basse. Par contre, dans le groupe témoin, la dystocie des épaules avait compliqué 12 accouchements, soit 1,5% de l’ensemble des accouchements et 1,9% des accouchements par voie basse. La différence étant significative avec un p à 0,04. Les manœuvres obstétricales réalisées étaient: 1) La manœuvre de Mc Roberts, chez 10 patientes; 2) La pression sus pubienne, chez 13 patientes; 3) La manœuvre de Jacquemier, chez 4 patientes.

Cette dystocie était survenue dans 48% des cas chez des macrosomes de poids de naissance supérieur ou égal à 4500 g (n=12) et dans 27% (n=15) chez des macrosomes de poids de naissance < 4500 g. Cette différence était aussi significative avec P < 0,05. Ce qui fait que la fréquence de la dystocie des épaules augmente avec l’augmentation du poids de naissance

Des traumatismes obstétricaux étaient observés chez 98 macrosomes et chez 75 nouveau-nés de poids normal. La fracture de la clavicule était plus fréquente dans le groupe des macrosomes que dans le groupe témoin. La différence était significative. Par contre, l’hématome sous cutané et la paralysie du plexus brachial étaient plus fréquents dans le groupe des macrosomes que dans le groupe témoin, sans que cette différence ne soit significative.

### Mortalité périnatale

Parmi les 820 naissances étudiées, 4 décès avant le 7^ème^ jour de vie étaient survenus soit un taux de mortalité précoce de 4,8‰ dans le groupe des macrosomes contre 3 décès dans le groupe témoin soit un taux de mortalité précoce de 3,7‰. Cette différence n’est pas significative.

## Discussion

L’incidence de la macrosomie ne cesse d’augmenter ces 50 dernières années. Dans notre étude, cette fréquence était de 10.9%. Elle se trouvait parmi les plus élevées dans la littérature [4–6]. De nombreuses études ont tenté d’identifier les facteurs de risques de la macrosomie. Toutefois, il y a beaucoup de controverse concernant ce sujet. L’âge maternel avancé, supérieur à 35 ans était rapporté dans plusieurs études comme étant un FDR [7, 8]; la prise du poids maternel dépassant 25% durant la grossesse était également reconnue en tant que facteur de risque indépendant de la macrosomie [9, 10].

Dans notre étude, on n’a pas pu étudier la prise de poids en tant que FDR vu que le poids pré gravidique n’était pas documenté dans les dossiers. L’obésité est également rapportée. Elle exerce une influence directe et significative sur le risque de macrosomie. Ce risque est indépendant de celui produit par le diabète; en effet, l’augmentation de l’IMC pré gravidique est directement corrélée à une augmentation du risque de macrosomie. Par ailleurs, Certaines études ont montré que l’obésité pré gravidique est plus prédictive de macrosomie que la prise de poids pendant la grossesse [11]. Cependant, l’étude de l’obésité maternelle en tant que FDR se heurte à l’absence de définition précise de la femme enceinte obèse [12].

D’autres études [9, 13] ont démontré que le diabète gestationnel est un facteur prédisposant à la macrosomie. Dans notre étude, cette association est significative (p=0,01); par contre d’autres auteurs [14] n’ont pas retrouvé cette liaison surtout si le diabète est bien équilibré. Une différence statistiquement significative était retrouvée entre le groupe des macrosomes et le groupe contrôle concernant la multiparité (p<0,001, les antécédents de macrosomie p<0,001) de mort fœtale in utéro (p<0,02) et la mort néonatale (p<0,01). Ainsi, Boulet et al [15] ont monté que l’antécédent de MFIU est le facteur de risque le plus important de macrosomie, avec une augmentation de plus en plus grande de l’importance de ce facteur de risque avec l’augmentation du grade de macrosomie.

Dans notre étude, le taux de grossesse à terme dépassé était de 26,9% dans le groupe macrosome versus 7% dans le groupe témoin. La différence était significative (p<0.001). Le sexe masculin était cité, par plusieurs études, comme étant un facteur de risque de macrosomie [16, 17]. Ainsi, des études à variables uniques que multiples ont montré que le sexe masculin est une variable prédictive de macrosomie. Le sexe ratio (Garçon/fille) était de 1.7 dans notre série.

Dans notre travail la césarienne était plus fréquente dans le groupe de macrosomes (24,6%) que le groupe contrôle (17,25%) avec DSS (p<0.001); mais notre étude comme plusieurs autres études [12] a montré que l’accouchement par voie basse (75,3%) était prédominant dans le groupe des macrosomes néanmoins, une aide instrumentale était plus pratiquée (5,6%) qu’on cas d’eutrophie (3,5%) (p=0,002). Une césarienne prophylactique était réalisée chez 16.6% de nos patientes. Cette attitude vise à réduire la morbidité fœtale [18]. 33.8% de nos césariennes prophylactiques étaient pour PFE ≥ 4500g. Alors que plusieurs études ont démontré que l’accouchement par voie basse est une alternative plus raisonnable à la césarienne élective. Le collège Américain de gynécologie obstétrique a suggéré la réalisation d’un accouchement par césarienne prophylactique pour toute suspicion de macrosomie avec PFE ≥ 5000g chez la femme non diabétique et à ≥ 4500g en cas de diabète. L’attitude dans notre service était de réaliser une Césarienne prophylactique en cas de siège et/ou utérus cicatriciel avec PFE ≥ 4000g; bien que pour de nombreux auteurs, la Macrosomie en elle même n’est pas une contre indication à l’épreuve utérine [19 ,20].

Quant à la gestion du travail chez les patientes porteuses de fœtus macrosomes.

Quant à la gestion du travail chez les patientes porteuses de fœtus macrosomes, certains auteurs [21, 22] ont trouvé que L’induction du travail a été associée à un risque plus élevé de césariennes et il n’y a pas lieu d’affirmer que l’induction du travail est une stratégie bénéfique [23]. Par ailleurs, l’allongement de la deuxième phase du travail est une caractéristique de l’accouchement d’un macrosome [24, 25]. Dans notre étude une phase active > 3h était observée dans 76,3% des cas de Macrosomie versus 46,7% dans le groupe contrôle (46,7%) (p<0,001). Le recours fréquent à la césarienne au cours du travail serait en rapport avec le défaut d’accommodation fœto-pelvienne et la mauvaise qualité des contractions en raison de la sur distension utérine [24]. Notre taux de césarienne au cours du travail était de 8% en cas de macrosomie. Il se situait parmi les plus faibles taux retrouvés dans la littérature [5, 6], et il serait en rapport avec une sélection très stricte des tentatives d’épreuves utérine.

Dans notre série on n’a déploré aucun cas de décès maternel. Cependant, la fréquence des complications maternelles étaient de 17%; dans les ¾ cas suite à un accouchement par voie basse. L’incidence de la morbidité maternelle dans notre série était un peu plus élevée que celle d’autre série ceci est du au fait que nous tenons compte de tous les cas d’hémorragie de la délivrance et d’inertie utérine aussi minime soit elle. L’augmentation du taux des complications maternelles avec l’augmentation du poids fœtale semble être due essentiellement à l’augmentation des lésions traumatiques et des hémorragies de la délivrance [8, 14]. La fréquence des lésions traumatiques génitales dans notre étude, évaluée à 2,9%, est comparable à celles retrouvées dans la littérature [5, 6, 14, 25] de même pour les complications infectieuses (2,2%). La fréquence de la mortalité périnatale était variable selon les auteurs de 0 à 6.2% [4, 24, 25]. Dans notre étude 4,8% des fœtus macrosomes peuvent succomber soit au cours de l’accouchement soit par dystocie des épaules irréductible, soit suite à une complication métabolique telle qu’une hypoglycémie ou une hypocalcémie sévère. On a constaté une différence statistiquement significative entre le groupe macrosome et le groupe contrôle concernant la dystocie des épaules, les traumatismes obstétricaux et l’hypoglycémie qui étaient plus élevés en cas de macrosomie. Dans notre série 85 nouveaux nés avaient nécessité une hospitalisation (10,3%).

## Conclusion

L’accouchement d’un fœtus macrosome est un accouchement à haut risque materno-fœtal. Les complications maternelles sont dominées par l’hémorragie de la délivrance et la rupture utérine alors que les complications fœtales sont dominées par la dystocie des épaules et les traumatismes obstétricaux. La prévention de la macrosomie fœtale et l’estimation exacte du poids de naissance ont longtemps été identifiées comme étant des objectifs essentiels dans la politique visant à diminuer la morbidité materno-fœtale. Une équipe pluridisciplinaire formée par un obstétricien chevronné, un néonatologiste et un médecin anesthésiste doit être présente dans la salle d’accouchement afin d’assister un tel accouchement et de prendre en charge précocement les éventuelles complications inhérentes à l’accouchement des macrosomes.

### Etat des connaissances actuelles sur le sujet

- L’accouchement d’un fœtus macrosome est un accouchement à haut risque materno-fœtal.

### Contribution de notre étude à la connaissance

- La prévention basé sur la détection des facteurs de risque de la macrosomie est primordial.

## Conflits d’intérêts

Les auteurs déclarent aucun conflit d'intérêts.
